# Impact of insomnia upon inflammatory digestive diseases and biomarkers: a two-sample mendelian randomization research on Europeans

**DOI:** 10.1186/s12876-024-03173-3

**Published:** 2024-02-21

**Authors:** Lei Dai, Yunyan Ye, Joseph Mugaanyi, Caide Lu, Changjiang Lu

**Affiliations:** 1 Department of Hepato-Pancreato-Biliary Surgery, Health Science Center, Ningbo Medical Centre Lihuili Hospital, Ningbo University, 1111 Jiangnan Road, Ningbo, 315040 Zhejiang China; 2 Department of Ophthalmology, Health Science Center, Ningbo Medical Centre Lihuili Hospital, Ningbo University, Ningbo, 315040 Zhejiang China

**Keywords:** Insomnia, Inflammatory digestive disease, Mendelian randomization, SNPs, Statistical association

## Abstract

**Background:**

A number of observational studies indicate that insomnia is linked to inflammatory digestive diseases (IDDs). However, the definite relationship between insomnia and IDDs remains unclear.

**Methods:**

We obtained the publicly available data from genome-wide association studies (GWAS) to conduct two-sample Mendelian randomization (MR) for association assessment. Five MR analysis methods were used to calculate the odds ratio (OR) and effect estimate, and the heterogeneity and pleiotropy tests were performed to evaluate the robustness of the variable instruments (IVs).

**Results:**

One exposure and twenty outcome datasets based on European populations were included in this study. Using the inverse variance weighted method, we found insomnia was closely correlated with esophageal ulcer (OR = 1.011, 95%CI = 1.004–1.017, *p* = 0.001) and abdominal pain (effect estimate = 1.016, 95%CI = 1.005–1.026, *p* = 0.003). Suggestive evidence of a positively association was observed between insomnia and duodenal ulcer (OR = 1.006, 95%CI = 1.002–1.011, *p* = 0.009), gastric ulcer (OR = 1.008, 95%CI = 1.001–1.014, *p* = 0.013), rectal polyp (OR = 1.005, 95%CI = 1.000-1.010, *p* = 0.034), haemorrhoidal disease (OR = 1.242, 95%CI = 1.004–1.535, *p* = 0.045) and monocyte percentage (effect estimate = 1.151, 95%CI = 1.028–1.288, *p* = 0.014). No correlations were observed among other IDDs, phenotypes and biomarkers.

**Conclusions:**

Our MR study assessed the relationship between insomnia and IDDs/phenotypes/biomarkers in depth and revealed potential associations between insomnia and ulcers of the esophagus and abdominal pain.

**Supplementary Information:**

The online version contains supplementary material available at 10.1186/s12876-024-03173-3.

## Introduction

 Inflammatory bowel disease (IBD) is a chronic, non-specific intestinal inflammatory illness that mostly includes Crohn’s disease (CD) and ulcerative colitis (UC) [1]. The incidence of IBD has increased globally in recent years, particularly in Europe and developing countries, posing a significant clinical challenge [2–5]. Although its probable causes are unknown, the immune impairment viewpoint gives a full picture of the disease’s multi-factor origin [6]. Intestinal bacterial disorders are one of the reasons for the development of IBD, as confirmed by the randomized, controlled PRASCO trial (using the metagenome method) [7]. In addition, therapeutic nutrition was considered to be associated with IBD alleviation [8, 9].

More and more studies show that interrupted sleep and irregular day and night rhythms can cause severe damage to the gastrointestinal tract [10]. A prospective cohort study demonstrated that sleep insufficiency and daytime napping significantly increased the risk of IBD [11]. This potentially indicates that the ability to fine-tune our intestinal barrier and the normal interaction between the mucous immune system and microorganisms is disrupted when the rhythm of the central nervous system is disturbed during the day and night. On the contrary, another retrospective cohort study including 48,799 IBD patients found that IBD patients were correlated with a higher incidence ratio of insomnia with a hazard ratio (HR) of 1.99 [12]. Moreover, several studies also found a relatively consistent conclusion that IBD might promote insomnia, which could be illustrated as symptoms like pain worsened sleep quality [13–15]. A questionnaire study showed that 81% of 312 respondents said they believed there was an interaction between sleep and IBD [15]. However, the association between insomnia and IBD still remains undefined. Current research may contain a selection bias by its nature, requiring us to interpret the results with caution. A randomized controlled study on this issue is urgently needed to confirm the potential relationship.

Additionally, peptic ulcer disease(PUD) [16, 17] and intestinal polyp [18] were also considered to be potentially correlated with sleep duration. All of these inflammatory digestive diseases (IDDs) cause physical and mental suffering and a high medical burden for patients. Hence, it is of great clinical value to explore the potential association between them and insomnia to benefit patients through a lifestyle shift.

Mendelian randomization (MR) analysis is an epidemiological statistical technique that uses observational data to estimate causality. It has been widely used in inferring the potential causal relationships between an exposure and an outcome, owing to its advantage of minimizing the influence of confounders by introducing genetic variants as instrumental variables (IVs) [19, 20]. Using the characteristics of random allocation of allelic genetic polymorphisms, MR has largely overcome the disadvantages of reverse causality bias and ethical issues [21].

Here we extend the concept of IBD to IDDs, which include 10 benign gastrointestinal inflammatory diseases. Two-sample MR was performed to assess the potential associations between insomnia and inflammatory diseases/phenotypes/biomarkers. In this study, we aim to answer two core questions: (1) whether there are potential relationships between insomnia and IDDs (positive/negative). (2) whether potential links exist between insomnia and IDD-related phenotypes and biomarkers.

## Materials and methods

### Study design

**Fig. 1:**
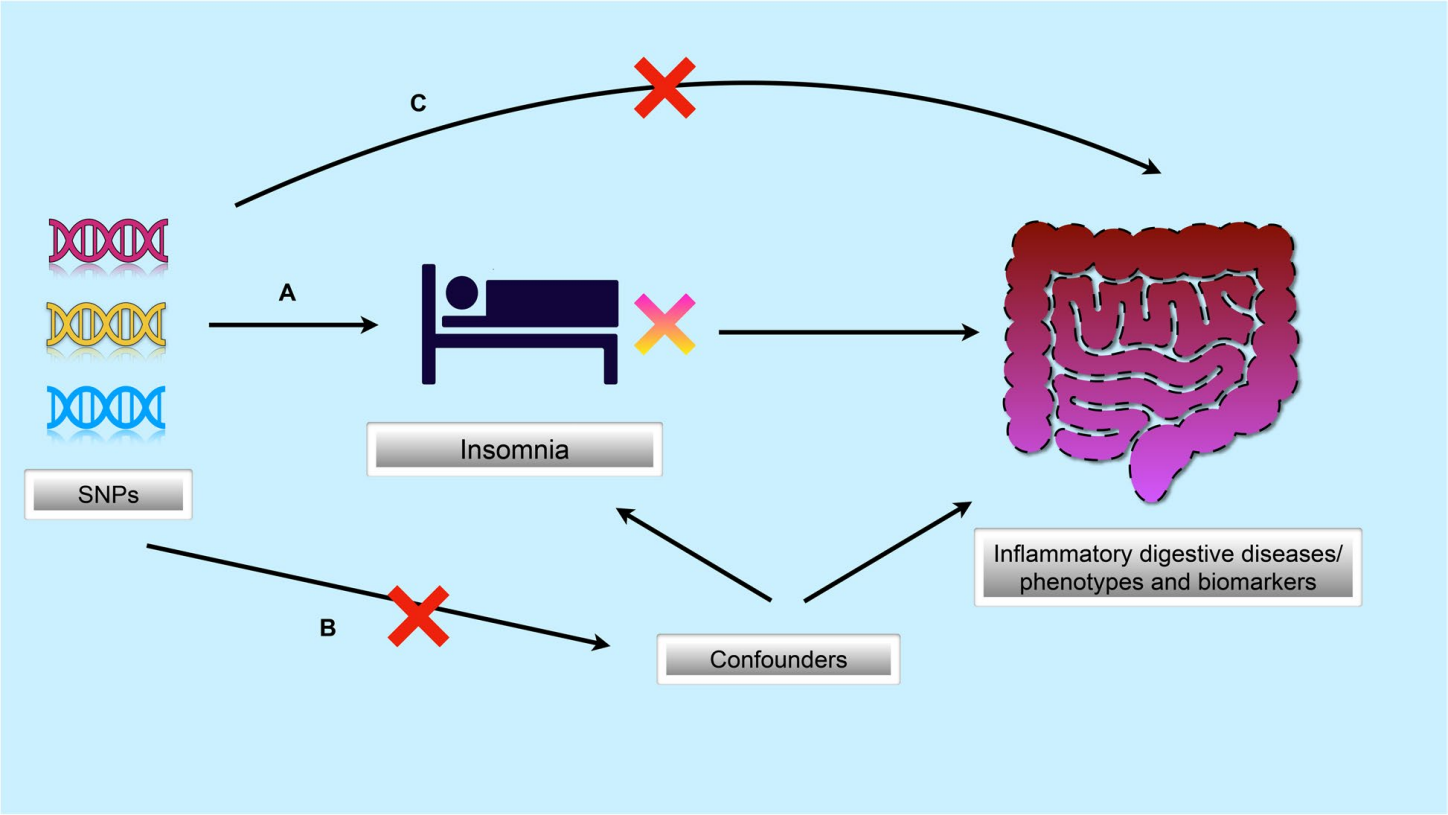
Three core assumptions of the MR study. **A** SNPs are closely associated with exposure (insomnia); **B** SNPs are independent of confounders; **C** SNPs only affect outcomes (inflammatory digestive diseases, phenotypes and biomarkers) through exposure of insomnia. MR, mendelian randomization; SNPs, single nucleotide polymorphisms

**Fig. 2:**
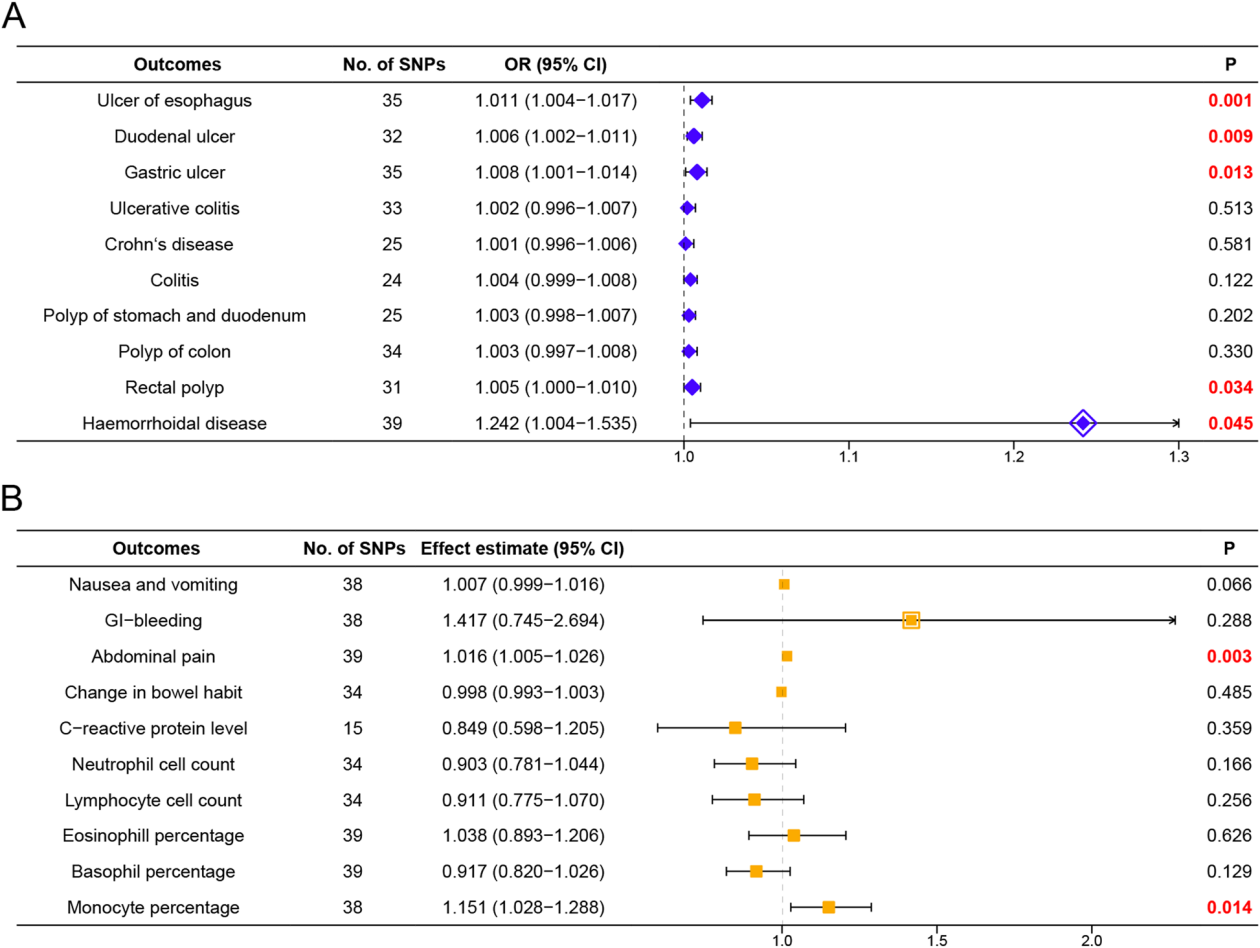
Associations of genetically predicted insomnia with inflammatory digestive diseases, phenotypes, and biomarkers. **A** Effect of insomnia on inflammatory digestive diseases using IVW analysis; **B** Effect of insomnia on inflammatory digestive phenotypes and biomarkers using IVW analysis. IVW, inverse-variance weighted; CI, confidence interval; OR, odds ratio; SNPs, single nucleotide polymorphisms

**Fig. 3:**
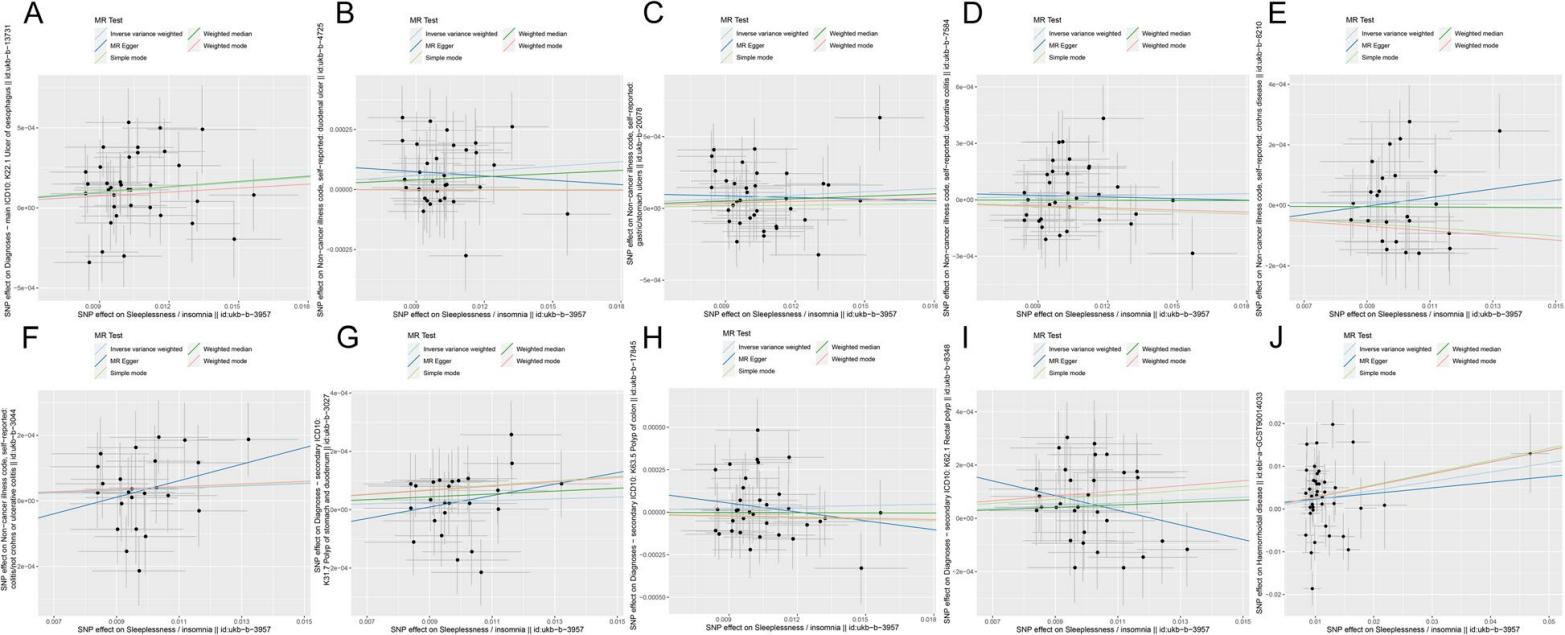
Scatter plot of the association of insomnia with inflammatory digestive diseases. **A** Ulcer of the esophagus; **B** Duodenal ulcer; **C** Gastric ulcer; **D** Ulcerative colitis; **E** Crohn’s disease; **F** Colitis; **G** Polyp of stomach and duodenum; **H** Polyp of the colon; **I** Rectal polyp; **J** Haemorrhoidal disease. Each dot represents an SNP, which is plotted with standard error bars by the estimate of SNP on individual sleep condition and the estimate of SNP on the risk of inflammatory digestive diseases. The slopes of the lines correspond to estimates calculated using each of the five approaches. SNP, single nucleotide polymorphism

The overview of study design and three core hypotheses for genetic IVs are demonstrated in Fig. 1: (1) Relevance hypothesis: single nucleotide polymorphisms (SNPs) are strongly correlated with insomnia (Fig. 1A); (2) Independence hypothesis: SNPs are independent of known confounders (Fig. 1B); (3) Exclusivity hypothesis: insomnia is the only approach for SNPs affecting IDDs/phenotypes/biomarkers (Fig. 1C) [22].

### Exposure and outcome data

The open genome-wide association study (GWAS) database, based on scalable and high-performance cloud data infrastructure, supports complete GWAS summary datasets and metadata for the public (https://gwas.mrcieu.ac.uk/) [23]. This research was conducted using published data from GWASs of related traits in European individuals (both males and females included). The GWAS dataset for sleeplessness/insomnia (*n* = 462,341) was obtained from the MRC-IEU Consortium of the UK Biobank, in which estimated the correlation between insomnia and SNPs [24]. Ulcer of esophagus (*n* = 463,010), Duodenal ulcer (*n* = 462,933), Gastric ulcer (*n* = 462,933), Ulcerative colitis (*n* = 462,933), Crohn’s disease (*n* = 462,933), Colitis (*n* = 462,933), Polyp of stomach and duodenum (*n* = 463,010), Polyp of colon (*n* = 463,010) and Rectal polyp (*n* = 463,010) were obtained from the MRC-IEU Consortium [23]. Haemorrhoidal disease was obtained from the results reported by Zheng et al. [25]. For inflammatory digestive phenotypes, Nausea and vomiting (*n* = 463,010), Abdominal pain (*n* = 463,010), and Change in bowel habit (*n* = 463,010) were obtained from MRC-IEU Consortium [23]. Gastrointestinal (GI)-bleeding (*n* = 215,956) was obtained from the FinnGen biobank. As for inflammatory digestive biomarkers, C-reactive protein (CRP) level (*n* = 204,402) was derived from the results revealed by Ligthart et al. [26]. Neutrophil cell count (*n* = 563,946) and Lymphocyte cell count (*n* = 563,946) were achieved from Blood cell consortium [27]. Eosinophil percentage (*n* = 349,861), Basophil percentage (*n* = 349,861) and Monocyte percentage (*n* = 349,861) from Neale Lab. All the datasets were collected by using the TwoSampleMR R package. Details of all the datasets were summarized in Table 1.

**Table 1:** Baseline characteristics of insomnia and inflammatory digestive diseases, phenotypes and biomarkers

Trait	GWAS ID	Year	Author	Population	Sample Size	Case (n)	Control (n)	SNP (n)
Sleeplessness/insomnia	ukb-b-3957	2018	Ben Elsworth	European	462,341	-	-	9,851,867
Ulcer of esophagus	ukb-b-13,731	2018	Ben Elsworth	European	463,010	3,251	459,759	9,851,867
Duodenal ulcer	ukb-b-4725	2018	Ben Elsworth	European	462,933	1,908	461,025	9,851,867
Gastric ulcer	ukb-b-20,078	2018	Ben Elsworth	European	462,933	3,329	459,604	9,851,867
Ulcerative colitis	ukb-b-7584	2018	Ben Elsworth	European	462,933	2,439	460,494	9,851,867
Crohn’s disease	ukb-b-8210	2018	Ben Elsworth	European	462,933	1,401	461,532	9,851,867
Colitis	ukb-b-3044	2018	Ben Elsworth	European	462,933	1,193	461,740	9,851,867
Polyp of stomach and duodenum	ukb-b-3027	2018	Ben Elsworth	European	463,010	1,233	461,777	9,851,867
Polyp of colon	ukb-b-17,845	2018	Ben Elsworth	European	463,010	2,437	460,573	9,851,867
Rectal polyp	ukb-b-8348	2018	Ben Elsworth	European	463,010	1,837	461,173	9,851,867
Haemorrhoidal disease	ebi-a-GCST90014033	2021	Zheng T	European	944,133	218,920	725,213	8,424,267
Nausea and vomiting	ukb-b-4554	2018	Ben Elsworth	European	463,010	6,773	456,237	9,851,867
GI-bleeding	finn-b-K11_GIBLEEDING	2021	NA	European	215,956	4,992	210,964	16,380,464
Abdominal pain	ukb-b-6223	2018	Ben Elsworth	European	463,010	11,925	451,085	9,851,867
Change in bowel habit	ukb-b-10,368	2018	Ben Elsworth	European	463,010	2,443	460,567	9,851,867
C-reactive protein level	ieu-b-35	2018	Ligthart, S	European	204,402	NA	NA	2,414,379
Neutrophil cell count	ieu-b-34	2020	Vuckovic, D	European	563,946	NA	NA	NA
Lymphocyte cell count	ieu-b-32	2020	Vuckovic, D	European	563,946	NA	NA	NA
Eosinophill percentage	ukb-d-30210_irnt	2018	Neale lab	European	349,861	NA	NA	13,586,283
Basophil percentage	ukb-d-30220_irnt	2018	Neale lab	European	349,861	NA	NA	13,586,283
Monocyte percentage	ukb-d-30190_irnt	2018	Neale lab	European	349,861	NA	NA	13,586,283

*GWAS* Genome-wide association study, *SNP* Single nucleotide polymorphism, *NA* Not available

### Ethics statement

The GWAS summary-level data are publicly available and approved by their corresponding ethical review boards. Ethics approval was exempted for our study.

### SNPs selection and validation

In the present research, SNPs linked with insomnia were chosen and confirmed as IVs if they fulfilled the three conditions listed below: (1) The genome-wide significance threshold level was defined as *p* < 5E-08; (2) The linkage disequilibrium of SNPs threshold was set at *r* ^2^ < 0.001 and *Kb* = 10,000 to avoid the bias caused by them [28]; (3) The *F* statistic was calculated to assess the strength of each IV. To mitigate the bias caused by a weak instrumental variable, each SNP included must satisfy the condition of *F-*value > 10 [29, 30]. The formula is as follows [31]:

$$F=(N-K-1)/K\times \frac{{R}^{2}}{1-{R}^{2}}$$

$${R}^{2}=2\times (1-MAF)\times MAF\times {\left(\frac{\beta }{SD}\right)}^{2}$$

$$SD=SE\times \sqrt{N}$$

Annotation: MAF: minor allele frequency = eaf.exposure; SE = se.exposure; $\beta$ = beta.exposure; N: no. of samples; K: no. of SNPs.

Secondly, PhenoScanner V2 (http://www.phenoscanner.medschl.cam.ac.uk/) was used to remove the SNPs of confounders related to the exposure and outcome [32, 33]. Thirdly, data harmonization was performed to align the effect alleles of IVs.

### Statistical analysis

To estimate the potential association between insomnia and different IDDs/phenotypes/biomarkers comprehensively, random/fixed-effects inverse variance weighting (R/F-IVW), MR Egger, Weighted median, Simple mode and Weighted mode were performed for sensitivity analyses. The Mendelian estimates of different validity assumptions can be obtained from the above methods [34, 35]. We adopted IVW as the primary analysis method to report the odds ratio (OR) with 95% confidence intervals (CI), owing to its remarkable performance on accurate estimates and SNPs validation [36]. Additionally, MR-Egger regression and IVW were utilized to assess the heterogeneity of IVs. We utilized the MR-Egger interception method to test for pleiotropy and kicked out outliers via the MR-PRESSO method [37]. We conducted a leave-one-out analysis to evaluate whether and which individual SNPs could affect the overall estimates disproportionately. The Bonferroni correction method [38] was used to safeguard against the effect of multiple tests. Instead of using a *p*-value threshold of 0.05, *p* < 0.005 (α = 0.05/10 outcomes), *p* < 0.0125 (α = 0.05/4 phenotypes) and *p* < 0.008 (α = 0.05/6 biomarkers) were considered to be statistically significant for inflammatory digestive outcomes, phenotypes and biomarkers, respectively. If the Bonferroni-corrected value < *p* < 0.05, potential evidence of correlation was indicated, which needs further validation. We implemented all statistical analyses and visualizations employing the “Two-Sample MR” package [27] in R (version 4.0.3).

## Results

### Selection and validation of IVs

After screening, 42 SNPs that correlated strongly with insomnia in individuals of European descent were identified as IVs. All of them were verified to meet the criteria for IVs, with an *F* value > 10 (summarized in Table 2).

**Table 2:** Single nucleotide polymorphisms used as instrumental variables in the Mendelian randomization analyses of insomnia

SNP	Chr	A1	A2	SE	Beta	MAF	F-statistics	Nearby gene	*P*-value
rs2803296	1	C	G	0.001	-0.009	0.544	33	CALML6	7.30E-09
rs12049261	1	C	G	0.002	0.011	0.293	47	RP11-478L17.1	6.80E-12
rs6690017	1	G	T	0.002	-0.010	0.409	46	DAB1	1.10E-11
rs2644128	1	G	C	0.001	0.011	0.548	51	NAV1	1.00E-12
rs4572538	2	T	C	0.002	-0.010	0.364	38	PABPC1P2	7.70E-10
rs56365214	2	A	C	0.002	-0.015	0.156	52	LINC01122	5.60E-13
rs4577309	2	G	A	0.001	-0.009	0.534	33	MFSD6	1.00E-08
rs12470989	2	G	A	0.002	-0.010	0.204	31	MAIP1	2.80E-08
rs113851554	2	T	G	0.003	0.047	0.057	199	MEIS1	2.90E-45
rs56093896	2	A	C	0.002	-0.012	0.214	47	IGKV1OR2-108	7.70E-12
rs2014830	3	T	C	0.002	-0.012	0.304	51	SEMA3F-AS1	8.90E-13
rs705219	3	A	T	0.002	0.013	0.887	33	RP11-384F7.2	1.20E-08
rs9845387	3	A	C	0.004	-0.022	0.040	33	LSAMP	7.10E-09
rs1988337	4	G	A	0.001	0.008	0.552	31	CCSER1	2.10E-08
rs11097861	4	G	A	0.002	0.010	0.716	37	RP11-729M20.1	1.10E-09
rs2604551	4	G	T	0.002	-0.008	0.640	30	RP11-665G4.1	4.70E-08
rs1592757	5	C	G	0.002	0.010	0.356	43	RP11-6N13.1	4.30E-11
rs7711696	5	T	G	0.002	0.011	0.305	48	SMAD5	4.10E-12
rs1430205	5	T	C	0.001	0.009	0.462	40	TMEM161B-AS1	2.10E-10
rs314280	6	G	A	0.001	0.010	0.547	42	LIN28B	7.30E-11
rs6975972	7	G	A	0.002	-0.009	0.579	36	C7orf50	2.00E-09
rs8180817	7	C	G	0.002	-0.010	0.431	44	FOXP2	2.70E-11
rs17151854	8	T	G	0.002	0.013	0.152	39	MSRA	3.80E-10
rs11790060	9	C	T	0.002	-0.010	0.331	43	RP11-165J3.6	5.80E-11
rs224032	10	A	G	0.001	0.008	0.550	32	ALDH7A1P4	1.80E-08
rs17709610	10	G	A	0.002	-0.010	0.298	37	ACTR1A	9.50E-10
rs2297787	10	A	T	0.003	-0.018	0.080	42	CNNM2	9.60E-11
rs72924721	11	T	C	0.003	0.016	0.073	33	CFL1	1.10E-08
rs10838708	11	A	G	0.002	-0.009	0.459	40	PSMC3	2.90E-10
rs68094047	12	T	C	0.002	0.010	0.251	36	MYO1H	1.70E-09
rs931221	12	A	T	0.002	0.011	0.237	37	RP11-788H18.1	1.30E-09
rs324017	12	C	A	0.002	-0.010	0.705	37	NAB2	1.40E-09
rs9570080	13	C	T	0.002	-0.011	0.344	45	RPP40P2	1.60E-11
rs6561715	13	A	T	0.002	-0.012	0.631	57	RP11-384G23.1	4.80E-14
rs1547630	13	A	G	0.002	0.009	0.652	34	SNORD44	5.80E-09
rs4886860	15	C	G	0.002	-0.012	0.767	45	PML	1.80E-11
rs11635495	15	C	T	0.001	0.009	0.512	40	IQCH-AS1	2.80E-10
rs2062113	16	C	T	0.002	-0.010	0.568	41	AC040163.1	1.60E-10
rs9894577	17	A	G	0.002	0.013	0.318	68	HEXIM1	1.30E-16
rs9906181	17	G	A	0.002	-0.009	0.688	31	KCNJ12	2.40E-08
rs11152363	18	A	G	0.002	0.016	0.186	66	TCF4	4.50E-16
rs56330606	19	G	A	0.002	0.009	0.379	37	ZNF585B	1.20E-09

*SNP* Single-nucleotide polymorphisms, *Chr* Chromosome, *A1* Effect allele, *A2* Other allele, *SE* Standard error, *MAF *Minor allele frequency

### MR sensitivity analysis

We assessed the potential associations between insomnia and inflammatory digestive diseases, phenotypes and biomarkers in people of European descent mainly using the IVW approach. The results showed that insomnia might be positively correlated with all IDDs at the genetic level, while no statistically significant association was found for ulcerative colitis, Crohn’s disease, colitis, polyp of colon and polyp of the stomach and duodenum (all *p* > 0.05). Based on the analysis, we speculated that there might be a potential relationship between insomnia and ulcer of the esophagus (OR = 1.011, 95%CI = 1.004–1.017, *p* = 0.001). However, only suggestive evidence of positive associations was observed in duodenal ulcer (OR = 1.006, 95%CI = 1.002–1.011, *p* = 0.009), gastric ulcer (OR = 1.008, 95%CI = 1.001–1.014, *p* = 0.013), rectal polyp (OR = 1.005, 95%CI = 1.000-1.010, *p* = 0.034) and haemorrhoidal disease (OR = 1.242, 95%CI = 1.004–1.535, *p* = 0.045) (Fig. 2A). For most IDDs, the results of MR-Egger and weighted-median analyses revealed approximate estimates of lower exactness (Table 3). No obvious evidence of horizontal pleiotropy was detected (all *p* > 0.05). Based on the heterogeneity test, the fixed-effects model was applied to most IDDs except haemorrhoidal disease (*p* = 1.04E-08) which adopted the random-effects model to alleviate the effect of heterogeneity (Table 3).

**Table 3:** Associations between genetically predicted insomnia and inflammatory digestive diseases in sensitivity analyses using the weighted-median and MR-Egger methods

Outcome	Weighted Median		MR-Egger		Heterogeneity		Pleiotropy	
	OR (95%CI)	*P*	OR (95%CI)	*P*	Q	*P*	Intercept	*P*
Ulcer of esophagus	1.011 (1.002–1.020)	**0.017**	1.011 (0.967–1.058)	0.623	41.84	0.167	-6.95E-06	0.977
Duodenal ulcer	1.004 (0.998–1.011)	0.203	0.994 (0.959–1.030)	0.740	26.64	0.690	1.29E-04	0.490
Gastric ulcer	1.006 (0.997–1.014)	0.201	0.996 (0.955–1.039)	0.865	36.15	0.368	1.20E-04	0.594
Ulcerative colitis	1.000 (0.992–1.007)	0.954	0.997 (0.962–1.033)	0.877	30.97	0.519	4.85E-05	0.798
Crohn’s disease	0.999 (0.992–1.007)	0.888	1.014 (0.965–1.066)	0.584	28.55	0.237	1.30E-04	0.618
Colitis	1.004 (0.997–1.010)	0.266	1.026 (0.986–1.067)	0.220	22.42	0.495	2.16E-04	0.286
Polyp of stomach and duodenum	1.005 (0.999–1.011)	0.117	1.020 (0.981–1.060)	0.331	21.16	0.629	1.68E-04	0.403
Polyp of colon	1.000 (0.992–1.007)	0.952	0.983 (0.947–1.020)	0.366	36.63	0.304	2.11E-04	0.293
Rectal polyp	1.005 (0.998–1.012)	0.203	0.973 (0.932–1.015)	0.214	30.82	0.425	3.33E-04	0.139
Haemorrhoidal disease	1.317 (1.071–1.619)	**0.009**	1.141 (0.568–2.294)	0.713	108.53	**1.04E-08**	1.00E-03	0.805

*CI* Confidence interval, *MR* Mendelian randomization, *OR* Odds ratio

For inflammatory digestive phenotypes, the IVW analysis demonstrated that insomnia potentially correlated with abdominal pain (effect estimate = 1.016, 95%CI = 1.005–1.026, *p* = 0.003). Additionally, genetically predicted liability to insomnia might be positively correlated with nausea and vomiting and GI-bleeding, although no statistically significant results were obtained. To our surprise, an inverse association between insomnia and change in bowel habit (effect estimate = 0.998, 95%CI = 0.993–1.003, *p* = 0.485) was observed, although the result was not statistically significant (Fig. 2B). As to inflammatory biomarkers, except for the suggestive evidence of a positive relationship between insomnia and monocyte percentage (effect estimate = 1.151, 95%CI = 1.028–1.288, *p* = 0.014), no statistically significant association between insomnia and other biomarkers was observed (all *p* > 0.05) (Fig. 2B). Consistent with above, MR-Egger and weighted-median analyses revealed approximate estimates but of lower exactness (Table 4). No obvious evidence of horizontal pleiotropy was detected (all *p* > 0.05). According to the heterogeneity test, the fixed-effects model was applied to inflammatory digestive phenotypes, while the random-effects model was applied to inflammatory digestive biomarkers (Table 4).

**Table 4:** Associations between genetically predicted insomnia and inflammatory digestive phenotypes and biomarkers in sensitivity analyses using the weighted-median and MR-Egger methods

Outcome	Weighted Median		MR-Egger		Heterogeneity		Pleiotropy	
	EE (95%CI)	*P*	EE (95%CI)	*P*	Q	*P*	Intercept	*P*
Nausea and vomiting	1.007 (0.995–1.019)	0.234	1.007 (0.979–1.035)	0.653	43.21	0.223	1.09E-05	0.947
GI-bleeding	1.639 (0.639–4.204)	0.304	1.561 (0.237–10.290)	0.646	27.99	0.857	-1.19E-03	0.915
Abdominal pain	1.009 (0.993–1.025)	0.274	1.006 (0.973–1.040)	0.717	37.49	0.493	1.15E-04	0.553
Change in bowel habit	1.000 (0.993–1.007)	0.998	1.006 (0.971–1.043)	0.731	28.20	0.705	-8.57E-05	0.651
C-reactive protein level	1.109 (0.789–1.559)	0.552	1.103 (0.109–11.179)	0.935	43.96	**6.01E-05**	-2.72E-03	0.826
Neutrophil cell count	0.962 (0.859–1.076)	0.496	1.165 (0.743–1.826)	0.512	169.74	**2.56E-20**	-3.09E-03	0.250
Lymphocyte cell count	0.892 (0.797–0.999)	**0.047**	0.853 (0.506–1.437)	0.554	215.17	**1.32E-28**	7.92E-04	0.797
Eosinophill percentage	1.112 (0.986–1.253)	0.084	0.910 (0.561–1.475)	0.704	152.87	**1.03E-15**	1.57E-03	0.577
Basophil percentage	0.895 (0.792–1.011)	0.075	0.722 (0.507–1.027)	0.078	84.24	**2.36E-05**	2.85E-03	0.170
Monocyte percentage	1.048 (0.936–1.173)	0.418	0.909 (0.640–1.293)	0.600	86.81	**6.99E-06**	2.82E-03	0.175

*CI* Confidence interval, *MR* Mendelian randomization, *EE* Effect estimate

**Fig. 4:**
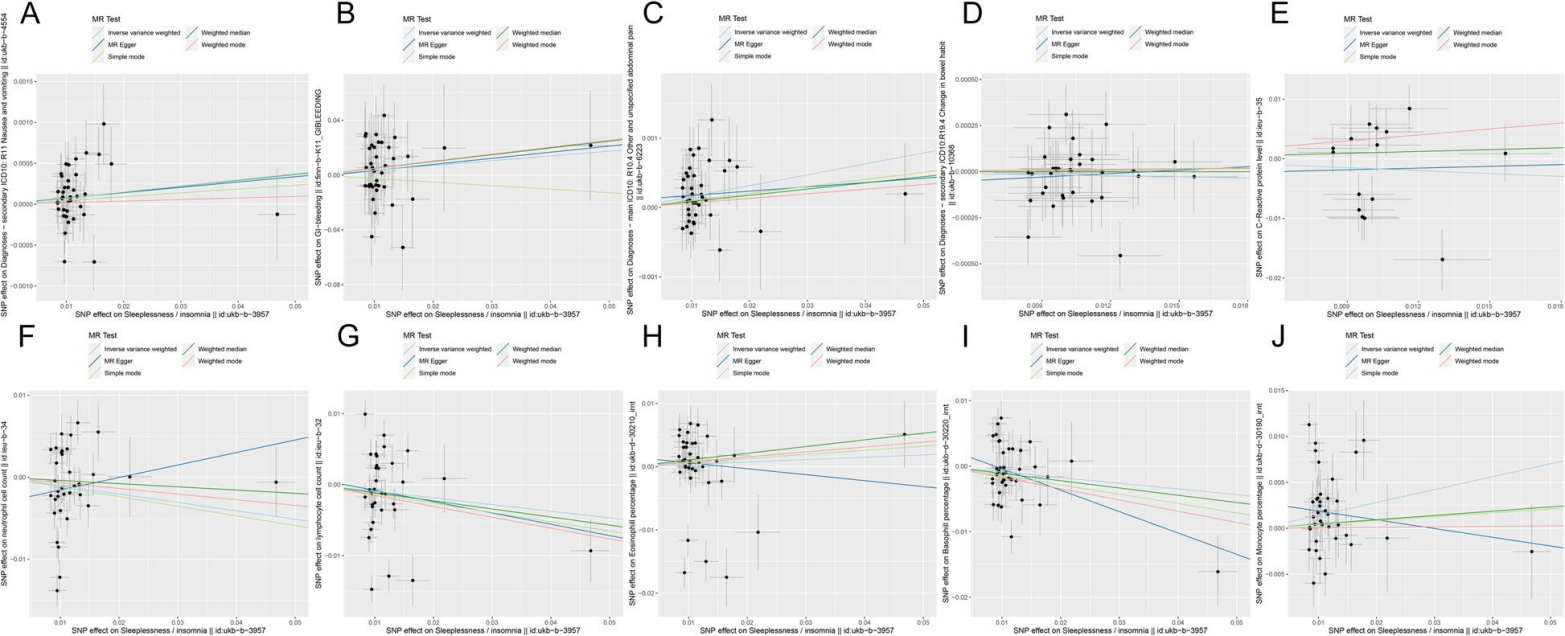
Scatter plot of the association of insomnia with inflammatory digestive phenotypes and biomarkers. **A** Nausea and vomiting; **B** GI-bleeding; **C** Abdominal pain; **D** Change in bowel habit; **E** C-reactive protein level; **F** Neutrophil cell count; **G** Lymphocyte cell count; **H** Eosinophill percentage; **I** Basophil percentage; **J** Monocyte percentage. Each dot represents an SNP, which is plotted with standard error bars by the estimate of SNP on individual sleep condition and the estimate of SNP on the risk of inflammatory digestive phenotypes and biomarkers. The slopes of the lines correspond to estimates calculated using each of the five approaches. GI, gastrointestinal; SNP, single nucleotide polymorphism

**Fig. 5:**
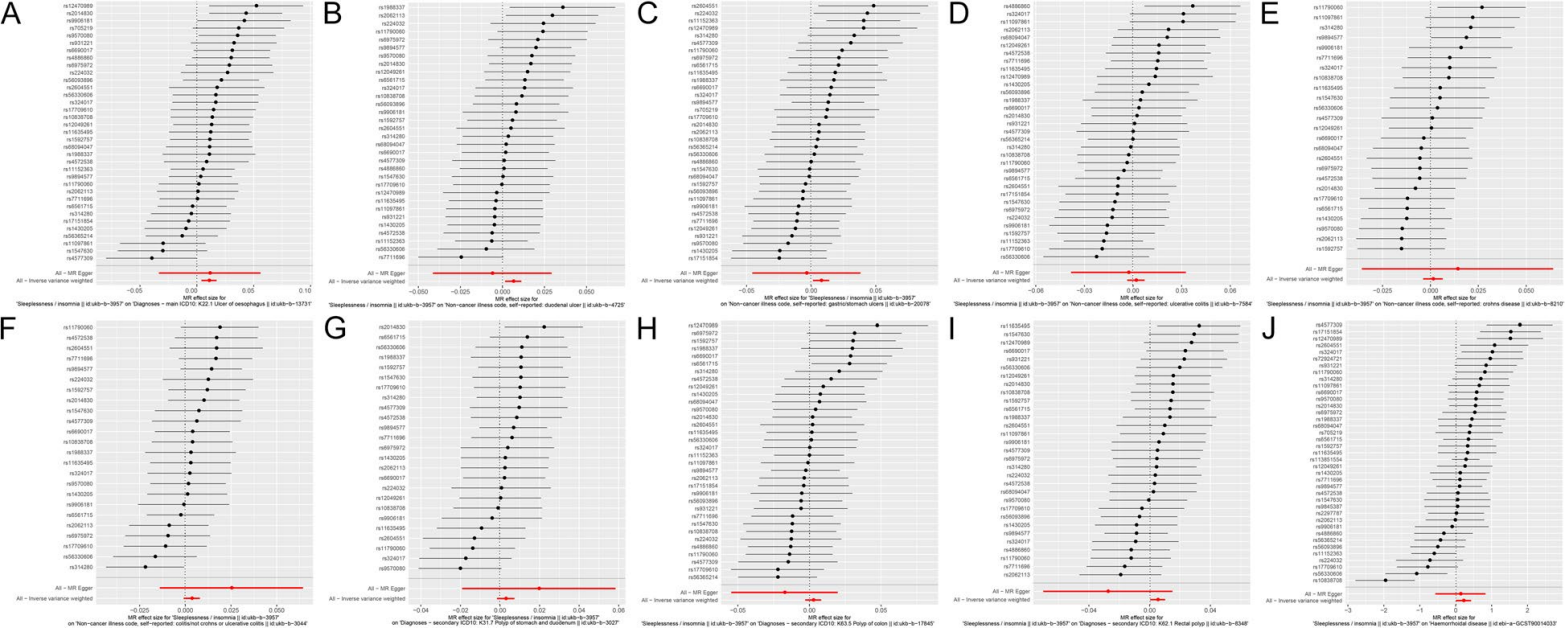
Forest plot of the association of insomnia with inflammatory digestive diseases. **A** Ulcer of the esophagus; **B** Duodenal ulcer; **C** Gastric ulcer; **D** Ulcerative colitis; **E** Crohn’s disease; **F** Colitis; **G** Polyp of stomach and duodenum; **H** Polyp of the colon; **I** Rectal polyp; **J** Haemorrhoidal disease. The dot and bar estimate the effect of each SNP related to insomnia on the risk of inflammatory digestive disease. SNP, single nucleotide polymorphism

**Fig. 6:**
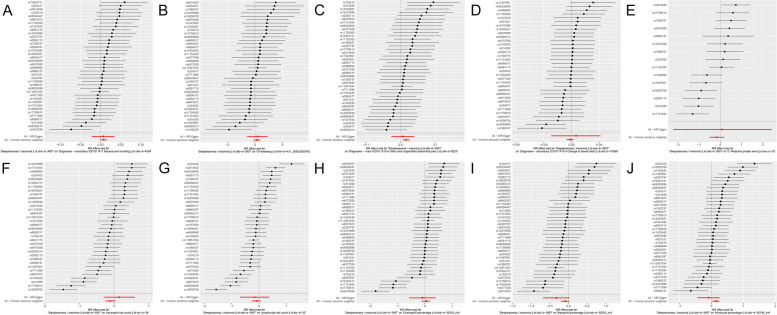
Forest plot of the association of insomnia with inflammatory digestive phenotypes and biomarkers. **A** Nausea and vomiting; **B** GI-bleeding; **C** Abdominal pain; **D** Change in bowel habit; **E** C-reactive protein level; **F** Neutrophil cell count; **G** Lymphocyte cell count; **H** Eosinophill percentage; **I** Basophil percentage; **J** Monocyte percentage. The dot and bar estimate the effect of each SNP related to insomnia on the risk of inflammatory digestive disease. GI, gastrointestinal; SNP, single nucleotide polymorphism

**Fig. 7:**
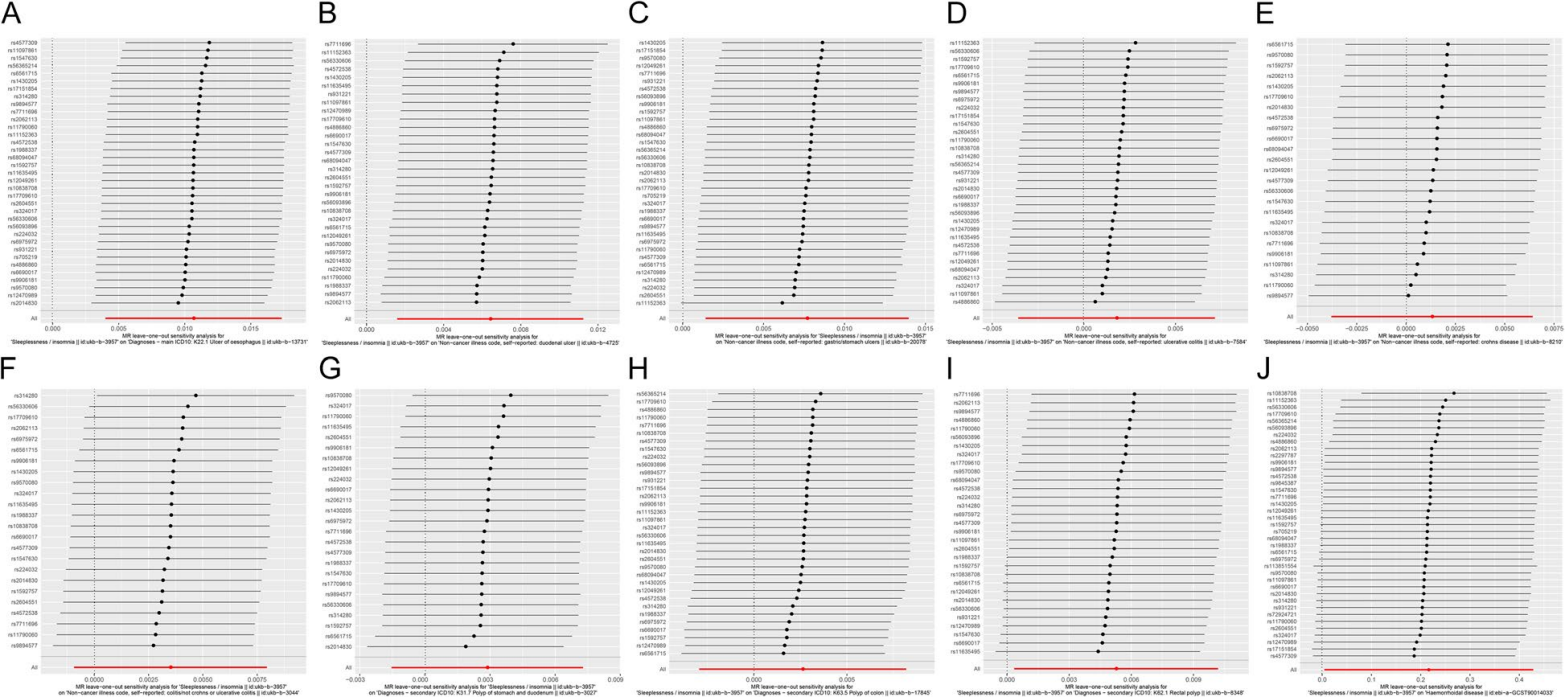
Leave-one-out sensitivity analysis of the association of insomnia with inflammatory digestive diseases. **A **Ulcer of the esophagus; **B** Duodenal ulcer; **C** Gastric ulcer; **D** Ulcerative colitis; **E** Crohn’s disease; **F** Colitis; **G** Polyp of stomach and duodenum; **H** Polyp of the colon; **I** Rectal polyp; **J** Haemorrhoidal disease. The dot and bar demonstrate the sensitivity of IV by removing SNPs one at a time. IV, instrument variable; SNPs, single nucleotide polymorphisms

**Fig. 8:**
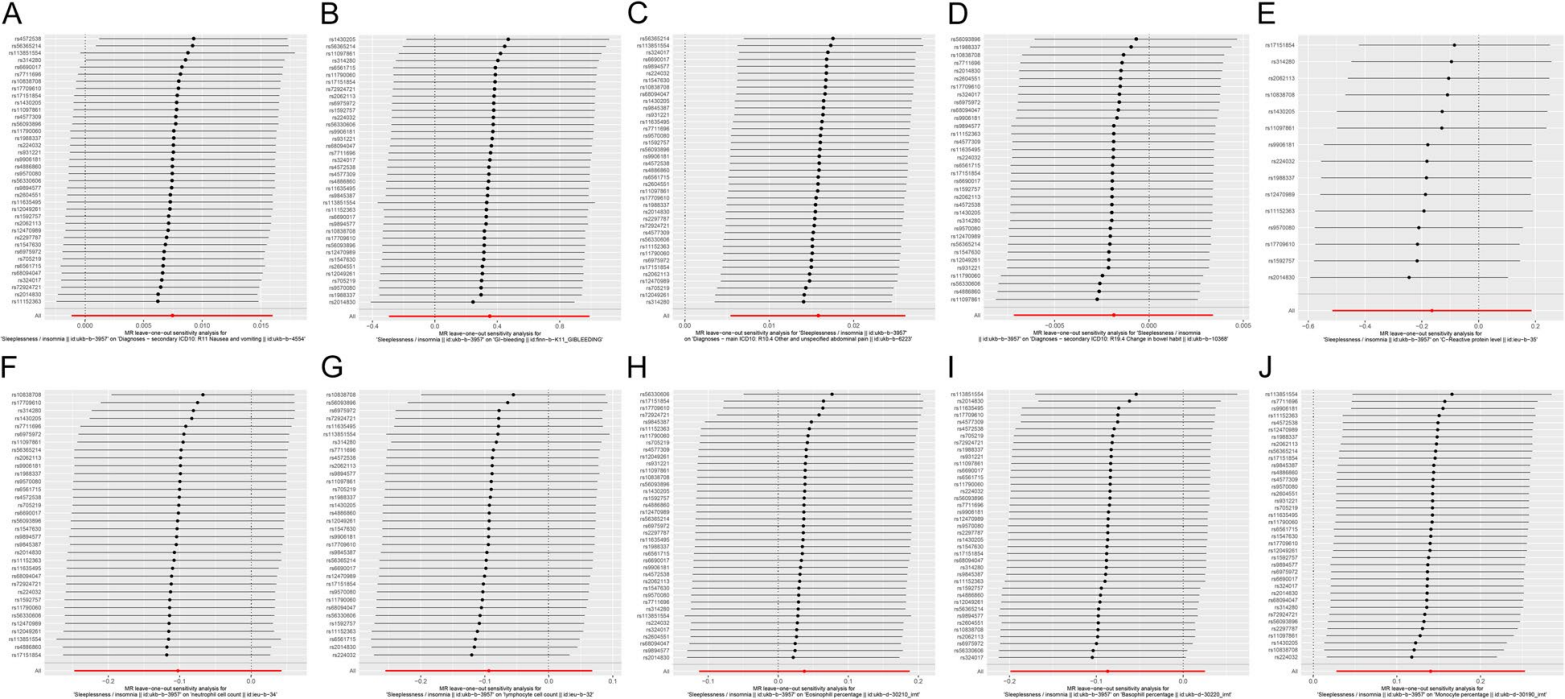
Leave-one-out sensitivity analysis of the association of insomnia with inflammatory digestive phenotypes and biomarkers. **A** Nausea and vomiting; **B** GI-bleeding; **C** Abdominal pain; **D** Change in bowel habit; **E** C-reactive protein level; **F** Neutrophil cell count; **G** Lymphocyte cell count; **H** Eosinophill percentage; **I** Basophil percentage; **J** Monocyte percentage. The dot and bar demonstrate the sensitivity of IV by removing SNPs one at a time. IV, instrument variable; SNPs, single nucleotide polymorphisms; GI, gastrointestinal

**Fig. 9:**
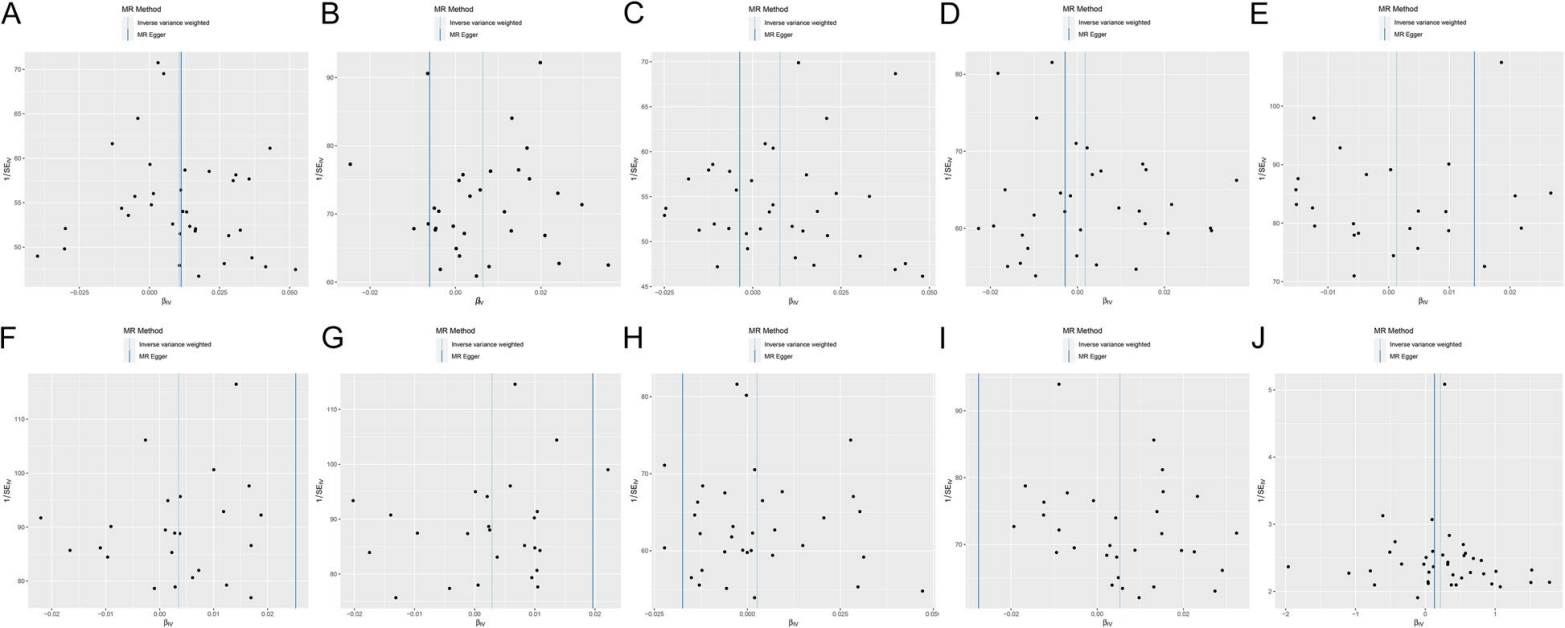
Funnel plot of the association of insomnia with inflammatory digestive diseases. **A** Ulcer of the esophagus; **B** Duodenal ulcer; **C** Gastric ulcer; **D** Ulcerative colitis; **E** Crohn’s disease; **F** Colitis; **G** Polyp of stomach and duodenum; **H** Polyp of the colon; **I** Rectal polyp; **J** Haemorrhoidal disease. Each black dot indicates a single nucleotide polymorphism

**Fig. 10:**
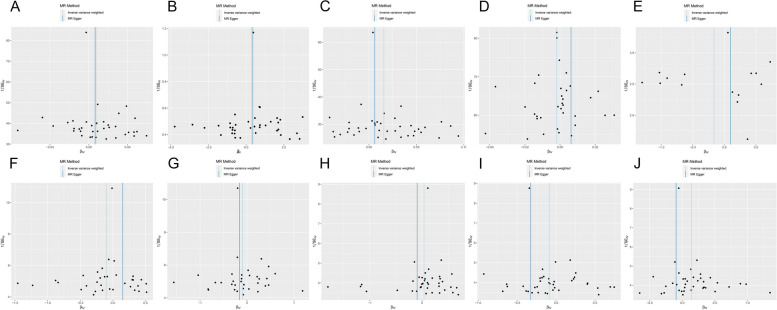
Funnel plot of the association of insomnia with inflammatory digestive phenotypes and biomarkers. **A** Nausea and vomiting; **B** GI-bleeding; **C** Abdominal pain; **D** Change in bowel habit; **E** C-reactive protein level; **F** Neutrophil cell count; **G** Lymphocyte cell count; **H **Eosinophill percentage; **I** Basophil percentage; **J** Monocyte percentage. Each black dot indicates a single nucleotide polymorphism. GI, gastrointestinal

Scatter plots of the association between insomnia and IDDs/phenotypes and biomarkers showed similar results (Figs. 3 and 4). Forest plot displayed each SNP’s influence on the associations between insomnia and IDDs/phenotypes and biomarkers (Figs. 5 and 6). For additional confirmation, the leave-one-out sensitivity analysis showed that no particular SNP altered the total estimates of IVs excessively, which was consistent with previous results (Figs. 7 and 8). The absence of horizontal pleiotropy was also confirmed by the funnel plot (Figs. 9 and 10).

## Discussion

For all we know, this MR study is the first one conducted to determine if insomnia is potentially associated with inflammatory digestive diseases, phenotypes and biomarkers. Our study originally extended the narrow-sense concept of IBD to the broad-sense concept of IDD, incorporating several approximate gastrointestinal disorders. Based on the Two-Sample MR analysis, we thoroughly evaluated the potential relationship between insomnia and inflammatory digestive diseases, phenotypes and biomarkers. The results disclosed that insomnia was positively associated with ulcers of the esophagus and abdominal pain. Furthermore, although only suggestive evidence was obtained, potential relationships were observed between insomnia and duodenal ulcer, gastric ulcer, rectal polyp, haemorrhoidal disease, and monocyte percentage.

PUD remains a common disease endangering public health worldwide [39], and there is no effective solution. Some research has claimed that an unhealthy lifestyle plays a critical role in PUD [40]. Sleeplessness, as one of the risk factors, was considered to be closely correlated with the development and recurrence of PUD [41–43]. To be consistent with this observational evidence, an MR study indicated a certain association between insomnia and PUD [16]. However, given the limitations of the previous studies, we conducted an MR analysis to investigate whether insomnia was closely correlated with PUD. Different from the previous MR study, we split PUD into ulcers of the esophagus, gastric, and duodenal regions for association assessments, respectively. When the three types of PUDs were treated as independent diseases, a potential association was observed between insomnia and them, respectively (all *p* < 0.05) (Fig. 2A). This result not only corroborated previous research but also provided more detailed and precise evidence. However, as components of IDDs, a definite association was solely observed between insomnia and ulcers of the esophagus. Only suggestive evidence existed for the potential relationship between insomnia and the other two PUDs, which might be attributed to stricter statistical thresholds. Although some studies revealed that digestive tract mucosa injury caused by immune, oxidative stress and circadian rhythm disturbances was the underlying mechanism of PUD induced by insomnia [10, 16], more randomized controlled trials (RCTs) and fundamental experiments are needed for further exploration and validation.

Many clinical studies revealed a correlation between sleeplessness and IBD, in which deficiencies in sleep duration and efficiency were strongly correlated with the progression of IBD [44–47]. Previous animal experiments [48, 49] and recent meta-analyses [50] have also confirmed the relationship between sleeplessness and IBD. Nevertheless, limited to the selection bias and potential confounders of the early studies, it is difficult to elucidate a causal relationship between them. Although our study failed to disclose any remarkable correlation between insomnia and IBD, the result was supported by a previous MR study [51]. Immune impairment and intestinal flora disruption caused by disturbed sleep rhythms are still widely recognized as an important trigger of IBD [6, 7, 10, 52], therefore, large-scale RCT/basic studies are urgently needed to further elucidate the intrinsic relationship between them.

Digestive tract polyps and haemorrhoidal diseases are common inflammatory proliferative diseases from a physiological angle. There is no available evidence to elucidate the association between sleep and these diseases. Although our study revealed no significant association between insomnia and digestive tract polyps for the first time, the ORs of these correlations were larger than 1 (Fig. 2A), indicating that insomnia might be a risk factor for these kinds of diseases. Besides, suggestive evidence was obtained from the potentially association between insomnia and haemorrhoidal diseases (Fig. 2A). However, these findings should be further validated in the future.

For inflammatory digestive phenotypes and biomarkers, the IVW method revealed that the genetic predisposition to insomnia was significantly correlated with abdominal pain and suggested evidence for a potential association between insomnia and monocyte percentage (Fig. 2B). Surprisingly, our analysis discovered no significant association between insomnia and other phenotypes and biomarkers. Such results might be attributed to the lack of high-specificity of these phenotypes and biomarkers for IDDs. Although specific pro-inflammatory cytokines (TNF-α, interleukin-1β and interleukin-6) were recognized to correlate with sleep and IBD closely [1, 53], we failed to obtain reliable evidence that insomnia had a potential relationship with the three inflammatory biomarkers due to the deficiency of the related GWAS dataset. As for the monocyte, a critical inflammatory-related immune cell, it is known to be closely correlated with insomnia [54, 55]. According to the studies, monocyte percentage is regulated by the circadian gene Bmal1 [56] and clock gene Arntl [57] and insomniac individuals have an increase in circulating monocytes. The findings of these studies provided credence to our research, but the definite association between insomnia and monocyte percentage still needs further validation.

There are several highlights to this study. The use of five MR analysis methods enhanced the reliability and comprehensiveness of the association assessment between exposure and outcome. And in essence, the MR study eliminated the potential confounders, reverse causality and other issues common in traditional epidemiological studies. Instead of a single SNP, multiple SNPs closely correlated with insomnia were used as IVs to decrease horizontal pleiotropy. Moreover, a homogenous population (European population) was used to reduce heterogeneity, which was prevalent when individuals of different races were included in genetic research. We further performed statistical corrections to make the results more robust.

However, our research still has some unavoidable limitations. First, several datasets with higher specificity were not included due to their small sample size. This might result in the absence of some potential associations, which need large-scale RCTs and basic studies for further elucidation. Second, although the population in our study was highly homogenous, whether the results could be generalized to individuals of various ancestry populations remains unknown. Moreover, some phenotypes/biomarkers may be expressed only during certain time periods of life, resulting in some potential associations being missed. Genetic pleiotropy cannot be completely ruled out, although we have done our best to minimize it.

## Conclusions

To sum up, our MR analysis revealed a well-established potential relationship between insomnia and IDDs/phenotypes/biomarkers including ulcer of the esophagus and abdominal pain, as well as suggestive evidence of a potential association among IDDs/phenotypes/biomarkers including gastric ulcer, duodenal ulcer, rectal polyp, haemorrhoidal disease and monocyte percentage. Sleep management and insomnia therapy may provide new insights into the prevention and treatment of IDDs and bring more benefits to patients.

### Supplementary Information

**Supplementary Material 1.**

## Data Availability

The original contributions presented in the study are included in the article/supplementary material, further inquiries can be directed to the corresponding author/s. All data and original files in our work are freely available under a ‘Creative Commons BY 4.0’ license. All methods were carried out in accordance with relevant guidelines and regulations.
